# Effects of glycyrrhizin on the pharmacokinetics of nobiletin in rats and its potential mechanism

**DOI:** 10.1080/13880209.2020.1751661

**Published:** 2020-04-16

**Authors:** Hao Wang, Lin Dong, Fangfei Qu, Huimin He, Wei Sun, Yuqing Man, Hongjie Jiang

**Affiliations:** a Department of Pharmacy, Yantai Affiliated Hospital of Binzhou Medical University, Yantai, Shandong, China; b Department of Special Inspection, Yantai Affiliated Hospital of Binzhou Medical University, Yantai, Shandong, China; c Department of Pharmacy, Binzhou Medical University Hospital, Binzhou, Shandong, China; d Department of Pediatrics, Yantai Affiliated Hospital of Binzhou Medical University, Yantai, Shandong, China

**Keywords:** Drug–drug interaction, CYP3A4, metabolism, P-gp, efflux

## Abstract

**Context:**

Both nobiletin (NBL) and glycyrrhizin (GL) have anti-inflammatory and antitumor properties. These agents may be co-administered in the clinic. However, the drug–drug interaction between them is not clear.

**Objective:**

The drug–drug interaction between GL and NBL was investigated, to clarify the effect of GL on the pharmacokinetics of NBL, and its main mechanism.

**Materials and methods:**

The pharmacokinetic profiles of oral administration of NBL (50 mg/kg) in Sprague-Dawley rats of two groups with six each, with or without pre-treatment of GL (100 mg/kg/day for 7 days), were investigated. The effects of GL on the metabolic stability and transport of NBL were also investigated through the rat liver microsome and Caco-2 cell transwell models.

**Results:**

The results showed that GL significantly decreased the peak plasma concentration (from 1.74 ± 0.15 to 1.12 ± 0.10 μg/mL) and the *t_1/2_* (7.44 ± 0.65 vs. 5.92 ± 0.68) of NBL, and the intrinsic clearance rate of NBL was increased by the pre-treatment with GL (39.49 ± 2.5 vs. 48.29 ± 3.4 μL/min/mg protein). The Caco-2 cell transwell experiments indicated that GL could increase the efflux ratio of NBL from 1.61 to 2.41.

**Discussion and conclusion:**

These results indicated that GL could change the pharmacokinetic profile of NBL, via increasing the metabolism and efflux of NBL in rats. It also suggested that the dose of NBL should be adjusted when co-administrated with GL in the clinic.

## Introduction

Nobiletin (NBL) is a naturally occurring polymethoxy-flavonoid, commonly found in citrus fruit peels (Nogata et al. 2006). Currently, NBL possesses a variety of physiological activities. It has been reported that NBL can induce apoptosis of human breast cancer cells (Liu et al. 2018), inhibit the viability of human renal carcinoma cells (Wei et al. 2019) and exert or not exert antileukemic effects (Chen et al. 2018). Combination of different drugs can affect the bioavailability of drugs and the anticancer effect. Combination of nobiletin and sorafenib exerted synergistic effects on the inhibition of metastatic prostate cancer cell viability (Guney Eskiler et al. 2019). As NBL is a substrate of CYP3A4, and its transportation mediated by *P-gp*, the drug–drug interaction might affect the pharmacokinetics of NBL, and then influence the bioavailability.

Glycyrrhizin (GL) is the primary ingredient of the liquorice root extract, which has been widely used in the treatment of various inflammatory diseases or as a tonifying herbal medicine (Zhang and Ye 2009). It also has anti-inflammatory, hepato-protective, and antitumor properties (Chen et al. 2017; Jia et al. 2017; Mu et al. 2017). It has been reported that GL can modulate the activity of CYP3A4 and *P-gp*, which might lead to drug–drug interaction (Tu et al. 2010; Zhao et al. 2018). Co-administration of different drugs is common in traditional Chinese medicine, and GL has been reported to inhibit the effect of other drugs (Chen et al. 2017; Yan et al. 2017; Zhao et al. 2018; Zhao et al. 2018).

In this study, the effect of GL on the pharmacokinetics of NBL was investigated. The *in vivo* pharmacokinetics of NBL in rats with or without pre-treatment with GL were determined using LC-MS/MS method. Additionally, the effects of GL on the metabolic stability and the transport of NBL were investigated using the rat liver microsome and the Caco-2 cell transwell models.

## Materials and methods

### Chemicals

GL (purity > 98%) and NBL (purity >98%) was obtained from Shanghai Standard Biotechnology Co., Ltd (Shanghai, China). Acetonitrile and methanol were purchased from Fisher Scientific (Fair Lawn, NJ, USA). Dulbecco’s modified Eagle’s medium (DMEM) and non-essential amino acid (NEAA) solution were purchased from Thermo Scientific Corp. (Logan, UT, USA). Foetal bovine serum (FBS) was obtained from GIBCO BRL (Grand Island, NY, US). Penicillin G (10,000 U/mL) and streptomycin (10 mg/mL) were purchased from Amresco (Solon, OH, USA). Hanks’ balanced salt solution (HBSS) was purchased from GIBCO (Grand Island, NY, USA). Ultrapure water was prepared with a Milli-Q water purification system (Millipore, Billerica, MA, USA). All other chemicals were of analytical grade or better.

### Animal experiments

Male Sprague-Dawley rats weighing 230–250 g were provided by the Animal Centre of Chinese Academy of Sciences (Shanghai, China). Rats were bred in a breeding room at 25 °C with 60 ± 5% humidity and a 12 h dark-light cycle. Tap water and normal chow were given *ad libitum*. All of the experimental animals were housed under the above conditions, for a three-day acclimation period and fasted overnight before the experiments. All experimental procedures and protocols were conducted in compliance with the National Institutes of Health Guide for the Care and Use of Laboratory Animal (NIH Publications No. 80-23, revised 1996) and the ethical principles of Binzhou Medical University Animal Experiment Committee (approval no. 2016002).

### In vivo pharmacokinetic study

To evaluate the effects of GL on the pharmacokinetics of NBL, the rats were divided into two groups of six animals each. The test group was pre-treated with GL at a dose of 100 mg/kg/day (dissolved directly in normal saline containing 0.5% methylcellulose at a concentration of 2 mg/mL) for 7 days before the administration of NBL, with the untreated group as control. Next, NBL was orally administered to rats by gavage at a dose of 50 mg/kg. The dosages used in the experiments were based on previous studies (Manthey et al. 2011; Guo et al. 2018; Sun et al. 2019). Blood samples (250 μL) were collected into heparinised tubes via the *oculi chorioideae* vein at 0.083, 0.33, 0.5, 1, 2, 4, 6, 8, 10, 12 and 24 h after the oral administration of NBL. The blood samples were centrifuged at 3500 rpm for 5 min. The plasma samples that were obtained were stored at −40 °C until analysis.

### LC-MS/MS determination of NBL

The determination of NBL was performed on Agilent 1290 series liquid chromatography system and an Agilent 6470 triple-quadruple mass spectrometer (Palo Alto, CA, USA). The HPLC/MS conditions and sample preparation were basically according to a validated HPLC method described by Zhang et al. (2019). The chromatographic analysis of NBL was performed on a Waters X-Bridge C18 column (3.0 × 100 mm, i.d.; 3.5 μm, USA) at room temperature (25 °C). The mobile phase was water (containing 0.1% formic acid) and acetonitrile (30:70, v:v) with isocratic elution at a flow rate of 0.2 mL/min, and the analysis time was 4 min.

The mass scan mode was positive MRM mode. The precursor ion and product ion are *m/z* 403.2→373.0 for NBL, and *m/z* 325.2→109.0 for IS. The collision energy for NBL and IS were 30 and 20 eV, respectively. The MS/MS conditions were optimized as follows: fragmentor, 110 V; capillary voltage, 3.5 kV; Nozzle voltage, 500 V; nebulizer gas pressure (N_2_), 40 psig; drying gas flow (N_2_), 10 L/min; gas temperature, 350 °C; sheath gas temperature, 400 °C; sheath gas flow, 11 L/min.

### Cell culture

The Caco-2 cell line was obtained from the American Type Culture Collection (Manassas, VA, USA), and it was performed according to the previous study (Liu et al. 2018). The Caco-2 cells were cultured in DMEM high glucose medium containing 15% FBS, 1% NEAA and 100 U/mL penicillin and streptomycin. The cells were cultured at 37 °C with 5% CO_2_. For transport studies, the cells at passage 40 were seeded on transwell polycarbonate insert filters (1.12 cm^2^ surface, 0.4 μm pore size, 12 mm diameter; Corning Co-star Corporation, MA, USA) in 12-well plates at a density of 1 × 105 cells/cm^2^. Cells were allowed to grow for 21 days. For the first seven days, the medium was replaced every two days, and then daily. The transepithelial electrical resistance (TEER) of the monolayer cells was measured using Millicell ERS-2 (Millipore Corporation, Billerica, MA, USA), and TEER exceeding 400 Ω·cm^2^ was used for the flux experiment. The integrity of the Caco-2 monolayers was confirmed by the paracellular flux of Lucifer yellow, which was less than 1% per hour. The alkaline phosphatase activity was validated using an Alkaline Phosphatase Assay Kit. The qualified monolayers were used for transport studies.

### Effects of GL on the absorption of NBL in the Caco-2 cell transwell model

Before the transport experiments, the cell monolayers were rinsed twice using warm (37 °C) Hanks’ balanced salt solution (HBSS), then the cells were incubated at 37 °C for 20 min. After preincubation, the cell monolayers were incubated with NBL in fresh incubation medium added on either the apical or basolateral side for the indicated times at 37 °C. The volume of incubation medium on the apical and basolateral sides was 0.5 mL and 1.5 mL, respectively, and a 100 μL aliquot of the incubation solution was withdrawn at the indicated time points from the receiver compartment and replaced with the same volume of fresh pre-warmed HBSS buffer. The inhibitory effects of *P-gp* inhibitors on the NBL flux by Caco-2 cells were investigated by adding 50 μM GL to both sides of the cell monolayers and preincubating the sample at 37 °C for 30 min. The permeability of NBL (2 μM) in all of the above conditions for both directions, i.e., from the apical (AP) side to the basolateral (BL) side and from the BL side to the AP side, was measured after incubation for 30, 60, 90 and 120 min at 37 °C. In addition, the efflux activity of *P-gp* was validated using typical *P-gp* substrate digoxin (25 μM).

The apparent permeability coefficient (*P_app_*) was calculated using the equation of Artursson and Karlsson:

$$P_{\mathrm{app}}=(\Delta Q/\Delta t)\times [1/(A\times C_{0})]$$

Where *P_app_* is the apparent permeability coefficient (cm/s), ΔQ/Δt (μmol/s) is the rate at which the compound appears in the receiver chamber, *C_0_* (μmol/L) is the initial concentration of the compound in the donor chamber and A (cm^2^) represents the surface area of the cell monolayer. Data were collected from three separate experiments, and each was performed in triplicate.

### Effects of GL on the metabolic stability of NBL in rat liver microsomes

Rat liver microsomes were used to investigate the effects of GL on the metabolism clearance of NBL, and the assay conditions and reaction mixtures were similar to those reported previously (Wang et al. 2017; Yan et al. 2017). In brief, 30 μL rat liver microsome (20 mg/mL), 12 μL NBL solution (100 μM) and 1113 μL PBS buffer (0.1 M, *pH* 7.4) were added to the centrifuge tubes on ice. There was a 5 min preincubation step at 37 °C before initiating the reaction by adding NADPH-generating system (45 μL) into the microsomal suspension. The effects of GL or ketoconazole (a positive CYP3A4 inhibitor) on the metabolic stability of NBL were investigated by adding 10 μM of GL or ketoconazole (12 μL, final concentration of 0.1 μM) to rat liver microsomes and preincubating them for 30 min at 37 °C, followed by the addition of NADPH-generating system. Aliquots of 100 μL were collected from the reaction volumes at 0.083, 0.167, 0.33, 0.5, 1, 2, 4, 8, 12 and 24 h after the addition of NBL, and 200 μL ice-cold acetonitrile containing esculin was added to terminate the reaction. All the experiments were performed in triplicate. The subsequent sample preparation method was the same as the plasma sample preparation, and the concentration of NBL was determined by LC-MS/MS.

The *in vitro half-life* (*t_1/2_*) was obtained using the equation: *t_1/2_ =* 0.693/*k*; V (μL/mg) = volume of incubation (μL)/protein in the incubation (mg); Intrinsic clearance (Clint) (μL/min/mg protein) = V × 0.693/*t_1/2_*.

### Data analysis

The pharmacokinetic parameters, including the area under the plasma concentration–time curve (*AUC*), maximal plasma concentration (*C_max_*), the time for the maximal plasma concentration (*T_max_*), and the mean residence time (*MRT*) were calculated using the DAS 3.0 pharmacokinetic software (Chinese Pharmacological Association, Anhui, China).

The differences between the mean values were analysed for significance using a one-way analysis of variance (ANOVA). Values of *p <* 0.05 were considered to be statistically significant.

## Results

### Effect of GL on the pharmacokinetics of NBL

**Figure 1.**
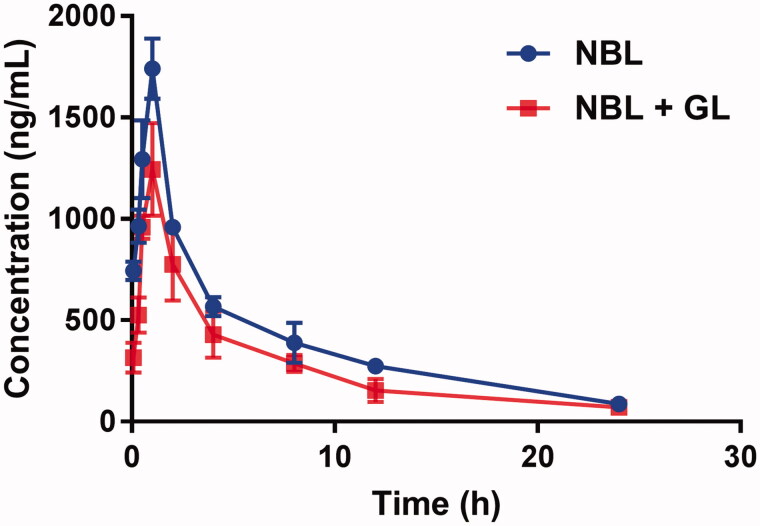
The pharmacokinetic profiles of NBL in rats (six rats in each group) after the oral administration of 50 mg/kg NBL with or without GL pre-treatment (100 mg/kg/day for 7 days). Each point represents the average ± S.D. of six determinations.

The mean plasma concentration–time curves of NBL with or without GL are shown in Figure 1, and the pharmacokinetic parameters were calculated using the noncompartmental method with the DAS 3.0 pharmacokinetic software (Chinese Pharmacological Association, Anhui, China). The pharmacokinetic parameters are summarized in Table 1.

**Table 1.** Pharmacokinetic parameters of NBL in rats after intragastrical administration of NBL (50 mg/kg; n = 6, Mean ± S.D.) with or without treatment of GL.

Parameter	Control	Pre-treatment of GL
T_max_ (h)	1 ± 0.08	1 ± 0.13
C_max_ (μg/mL)	1.74 ± 0.15	1.12 ± 0.10*
t_1/2_ (h)	7.44 ± 0.65	5.92 ± 0.48*
AUC _(0–t)_ (mg·h/L)	9.39 ± 1.02	5.95 ± 0.57*
MRT (h)	7.24 ± 0.69	6.36 ± 0.76*
CLz/F(L/h/kg)	4.88 ± 0.57	7.90 ± 0.40*

*p < 0.05 indicate significant differences from the control.

As shown in Table 1, the peak plasma concentration (*C_max_*) of NBL was 1.74 ± 0.15 μg/mL after oral administration of 50 mg/kg NBL. When the rats were pre-treated with GL (100 mg/kg) for 7 days, the *C_max_* decreased to 1.12 ± 0.10 μg/mL, and the difference was significant (*p* < 0.05). Additionally, the *AUC_0–t_* decreased significantly from 9.39 ± 1.02 to 5.95 ± 0.57 mg/h/L (*p* < 0.05). The decrease of *C_max_* and *AUC_0–t_* indicated that GL declined the concentration of NBL in the blood plasma. In the meantime, the half-life (*t_1/2_*) of NBL was shortened from 7.44 ± 0.65 to 5.92 ± 0.48 h, and the mean residence time (*MRT*) also decreased (7.24 ± 0.69 *vs.* 6.36 ± 0.76 h), and the difference was significant (*p* < 0.05). These results indicated that GL decreased the system exposure of NBL.

### Effects of GL on the bidirectional transport of NBL across Caco-2 cells

**Figure 2.**
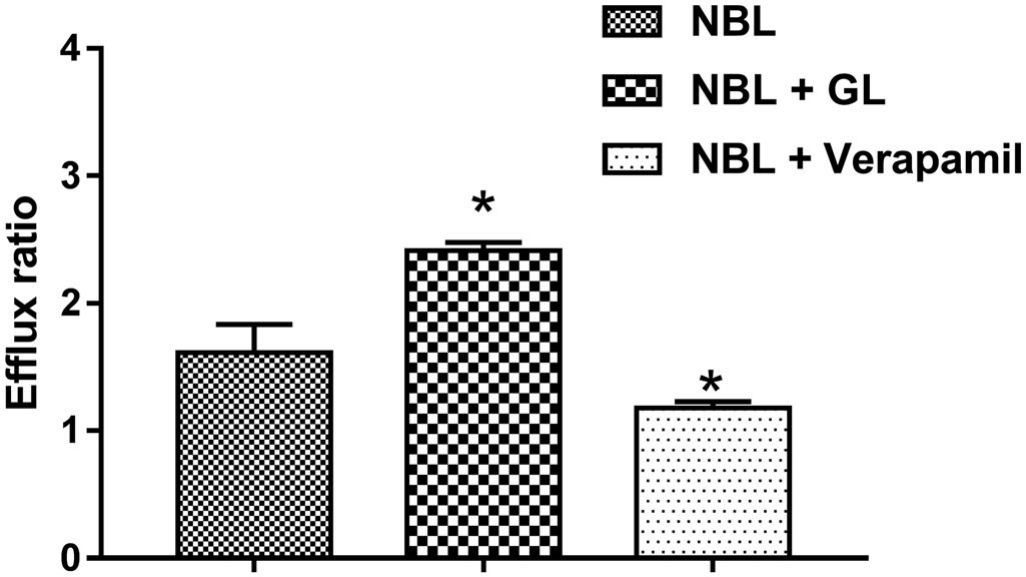
Effects of GL or verapamil on the efflux ratio of NBL in the Caco-2 cell monolayer model. Each point represents the average ± S.D. of three determinations. *p < 0.05 indicates significant differences from the triptolide group.

The effect of GL on the transport of NBL was investigated in the Caco-2 cells *in vitro* model. The *P-gp* inhibitor verapamil was employed to validate the efflux activity of *P-gp*. The efflux ratio of digoxin was 10.35 and it was suspended when verapamil was present. Therefore, the efflux activity of *P-gp* was qualified for the experiment. From the results of the *in vitro* Caco-2 cell model, the *P_appAB_* and *P_appBA_* value of NBL was 1.61 ± 0.15 × 10^−7^ and 2.58 ± 0.21 × 10^−7 ^cm/s, respectively. The *P_appBA_* value was much higher than *P_appAB_*, with the efflux ratio of 1.61, which indicated the efflux transporters might be involved in the transport of NBL. As shown in Figure 2, the efflux ratio significantly increased to 2.41 (*p* < 0.05), in presence of 50 μM GL, with the *P_appAB_* and *P_appBA_* value of 1.49 ± 0.13 × 10^−7^ and 3.58 ± 0.36 × 10^−7 ^cm/s, respectively. On the contrary, the efflux ratio decreased from 1.61 to 1.18, in the presence of verapamil (*p* < 0.05). These results indicated that *P-gp* was involved in the transport of NBL, and GL could enhance the efflux of NBL.

### Effects of GL on the metabolic stability of NBL in rat liver microsomes

The effect of GL on the metabolic stability of NBL was investigated in rat liver microsomes. The metabolic half-life (*t_1/2_*) was 35.1 ± 1.9 min, and it was shortened to 28.7 ± 2.4 min, in the presence of GL, the difference was significant (*p* < 0.05). Additionally, GL increased the intrinsic clearance rate of NBL from 39.49 ± 2.5 to 48.29 ± 3.4 μL/min/mg protein. These results indicated that the metabolism of NBL in rat liver microsomes was enhanced by GL, which is consistent with the results of the pharmacokinetics experiment.

## Discussion

NBL has been reported had several biological activities including anti-inflammatory, antiatherogenic, neuroprotective, neurotrophic, and antitumor properties. Currently, combination of different drugs can improve the efficiency of the treatment. GL has been widely used in the treatment of various diseases, and it is always co-administrated with other drugs in traditional Chinese medicine. Therefore, the interaction between NBL and GL was studied in this paper. It is worth to mention that except for the effect of GL on the pharmacokinetics of NBL, we also focussed on the effect on its transport.

The results showed that GL decreased the system exposure of NBL, and the administration of GL enhanced the metabolism of NBL, as the *C_max_* and *AUC_(0–t)_* significantly increased in the presence of GL. Meanwhile, the *t_1/2_* declined and the oral clearance of NBL increased, with the pre-treatment of GL. Additionally, the results were verified by the experiments in rat liver microsomes. In rat liver microsomes, the half-life of NBL was shortened with the value decreased from 35.1 ± 1.9 to 28.7 ± 2.4 min, and the intrinsic clearance rate increased, after incubation with GL. All these results indicated that GL enhanced the metabolism of NBL. There are several studies investigated the effect of GL on the pharmacokinetics of other drugs got similar results. GL decreased the system exposure of asiatic acid by inducing the activity of CYP450 enzymes (Guo et al. 2018), and GL increased the clearance rate of paeoniflorin via inducing the activity of CYP3A4 (Sun et al. 2019). The co-administration of GL and celastrol could decrease the plasma concentration of celastrol, due to the enhancement GL exerted on the activity of CYP3A4 (Yan et al. 2017).

We also investigated the effect of GL on the transport of NBL in the Caco-2 cells transwell model. It has reported that *P-gp* is involved in the transport of NBL (Kimura et al. 2014), and our results were consistent with previous studies that the efflux of NBL was much higher than its influx. The administration of GL accelerated the efflux of NBL, with the *P_appBA_* and the efflux ratio significantly increased. There are many studies have reported that GL could induce the activity of *P-gp,* and the transport of the drugs mediated by *P-gp* was induced by the administration of GL (Tai et al. 2014; Zhao et al. 2018). According to previous studies, NBL is the substrate of CYP3A4 (Koga et al. 2011), where GL can make induced effect (Tu et al. 2010), and our results indicated the transport of NBL involved *P-gp*. We inferred that the effect of GL on the pharmacokinetics profile was contributed to the induction of CYP3A4 or *P-gp*.

In summary, the co-administration of NBL and GL would influence the pharmacokinetic profile and the transport of NBL. GL exerted positive effects on the activity of CYP3A4 and *P-gp*, which are related to the metabolism and transport of NBL. Therefore, the metabolism and the efflux of NBL was enhanced by GL.

## Conclusions

From the above results, we conclude that GL decreased the system exposure of NBL through inducing the activity of CYP3A4, and due to the positive effect of GL on the activity of *P-gp*, the transport of NBL was enhanced. Although only one dose of GL and NBL was investigated in this study, these results also remind us that the dose of NBL should be adjusted when NBL and GL are co-administered in the clinic.
